# Genome-wide analysis of host-chromosome binding sites for Epstein-Barr Virus Nuclear Antigen 1 (EBNA1)

**DOI:** 10.1186/1743-422X-7-262

**Published:** 2010-10-07

**Authors:** Fang Lu, Priyankara Wikramasinghe, Julie Norseen, Kevin Tsai, Pu Wang, Louise Showe, Ramana V Davuluri, Paul M Lieberman

**Affiliations:** 1 The Wistar Institute, Philadelphia, PA 19104, USA; 2 Beth Israel Deaconess Medical Center, Boston MA, USA

## Abstract

The Epstein-Barr Virus (EBV) Nuclear Antigen 1 (EBNA1) protein is required for the establishment of EBV latent infection in proliferating B-lymphocytes. EBNA1 is a multifunctional DNA-binding protein that stimulates DNA replication at the viral origin of plasmid replication (OriP), regulates transcription of viral and cellular genes, and tethers the viral episome to the cellular chromosome. EBNA1 also provides a survival function to B-lymphocytes, potentially through its ability to alter cellular gene expression. To better understand these various functions of EBNA1, we performed a genome-wide analysis of the viral and cellular DNA sites associated with EBNA1 protein in a latently infected Burkitt lymphoma B-cell line. Chromatin-immunoprecipitation (ChIP) combined with massively parallel deep-sequencing (ChIP-Seq) was used to identify cellular sites bound by EBNA1. Sites identified by ChIP-Seq were validated by conventional real-time PCR, and ChIP-Seq provided quantitative, high-resolution detection of the known EBNA1 binding sites on the EBV genome at OriP and Qp. We identified at least one cluster of unusually high-affinity EBNA1 binding sites on chromosome 11, between the divergent FAM55 D and FAM55B genes. A consensus for all cellular EBNA1 binding sites is distinct from those derived from the known viral binding sites, suggesting that some of these sites are indirectly bound by EBNA1. EBNA1 also bound close to the transcriptional start sites of a large number of cellular genes, including HDAC3, CDC7, and MAP3K1, which we show are positively regulated by EBNA1. EBNA1 binding sites were enriched in some repetitive elements, especially LINE 1 retrotransposons, and had weak correlations with histone modifications and ORC binding. We conclude that EBNA1 can interact with a large number of cellular genes and chromosomal loci in latently infected cells, but that these sites are likely to represent a complex ensemble of direct and indirect EBNA1 binding sites.

## Introduction

Epstein-Barr virus (EBV) is a human lymphotropic gammaherpesvirus associated with a spectrum of lymphoid and epithelial cell malignancies, including Burkitt's lymphoma, Hodgkin's disease, nasopharyngeal carcinoma, and post-transplant lymphoproliferative disease (reviewed in [1,2]). EBV establishes a long-term latent infection in human B-lymphocytes where it persists as a multicopy episome that periodically may reactivate and produce progeny virus. During latency the EBV genome expresses a limited number of viral genes that are required for viral genome maintenance and host-cell survival. The viral gene expression pattern during latency can vary depending on the cell type and its proliferative capacity (reviewed in [3,4]). Among the latency genes, EBNA1 is the most consistently expressed in all forms of latency and viral-associated tumors. EBNA1 is required for the establishment of episomal latent infection and for the long-term survival of latently infected cells.

EBNA1 is a nuclear phosphoprotein that binds with high-affinity to three major DNA sites within the EBV genome [5](reviewed in [6]). At OriP, EBNA1 binds to each of the 30 bp elements of the family of repeats (FR), and to four 18 bp sequences within the dyad symmetry (DS) element. EBNA1 binding to OriP is essential for plasmid DNA replication and episome maintenance, and can also function as a transcriptional enhancer of the C promoter (Cp) [7,8]. At the Q promoter (Qp), EBNA1 binds to two 18 bp sequences immediately downstream of the transcriptional start site, and functions as an inhibitor of transcription initiation and mRNA accumulation [9]. EBNA1 binds directly to DNA through its C-terminal DNA binding domain [5,10]. The structure of the EBNA1 DNA binding domain has been solved by X-ray crystallography and was found to have structural similarity to papillomavirus E2 protein DNA binding domain [11,12]. In addition to direct DNA binding through the C-terminal domain, EBNA1 tethers the EBV genome to metaphase chromosomes through its amino terminal domain [13,14]. The precise chromosomal sites, proteins, or structures through which EBNA1 attaches during metaphase are not completely understood [14-16].

Recent studies have revealed that EBNA1 can bind to and regulate numerous cellular gene promoters [17,18]. Others have identified cellular phenotypes, like genomic instability, and the genes associated with genomic instability, to be regulated by ectopic expression of EBNA1 in non-EBV infected Burkitt lymphoma cell lines [19]. Overexpression of the EBNA1 DNA binding domain, which functions as a dominant negative in EBV infected cells, can inhibit cell viability in uninfected cells, suggesting that EBNA1 binds to and regulates cellular genes important for cell survival [20]. In more recent studies, EBNA1 binding was examined at a subset of cellular sites using predicted promoter arrays. However, EBNA1 is likely to bind to other regions of the cellular chromosome that may be important for long-distance enhancer-promoter interactions, as well as for regulation of chromatin structure and DNA replication. To explore these additional possible functions of EBNA1, we applied Solexa-based deep sequencing methods to analyze the genome-wide interaction sites of EBNA1 in latently infected Raji Burkitt lymphoma cells. Our results corroborate previous studies that demonstrate multiple cellular promoter binding sites for EBNA1, and extend these studies to reveal numerous EBNA1 binding sites not closely linked to a promoter start site. We conclude that EBNA1 has the potential to function as a global regulator of cellular gene expression and chromosome organization, similar to its known function in the EBV genome.

## Results

### ChIP-Seq Analysis of EBV and human genomes

**Figure 1:**
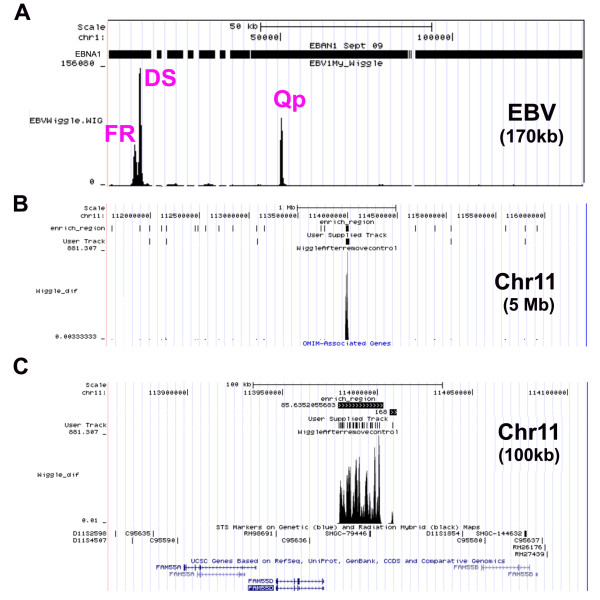
**Example of ChIP-Seq data on EBV genome and host-cell chromosome 11 EBNA1 binding site cluster**. The UCSC genome browser was used to map EBNA1 ChIP-Seq peak files and enrichment beds to the EBV genome (panel A) or human chromosome 11 FAM55B and D intergenic region at 1 MB (panel B) or 100 kB (panel C) resolution. Wiggle files show the fold enrichment calculated as EBNA1 over IgG, and the track count for EBNA1. Peaks for family of repeats (FR), dyad symmetry (DS), and Q promoter (Qp) are indicated in red for the EBV genome (A).

Raji Burkitt lymphoma cells were selected for EBNA1-ChIP-Seq experiments because they maintain a stable copy number of EBV episomes, and because the genomes are incapable of lytic replication (due to a mutation in BALF2), which might complicate ChIP analysis. Anti-EBNA1 monoclonal antibody and IgG control ChIP DNA was analyzed by Solexa-Illumina based deep sequencing methods. Sequence reads were mapped to the EBV or human genomes using the UCSC genome browser http://genome.ucsc.edu/cgi-bin/hgTracks, and a fold enrichment for EBNA1 relative to IgG control antibodies was calculated. A summary of the sequencing reads mapped to the human and viral genome is presented in Table 1. The EBNA1 enriched peaks that mapped to the EBV genome are shown in Figure 1A. We found three major peaks for EBNA1 mapping to the FR, DS and Qp region, as were predicted from earlier genetic and biochemical studies of EBNA1 binding to EBV DNA. No other regions were identified, indicating that these sites are likely to represent the major binding sites of EBNA1 in Raji genomes *in vivo*. Interestingly, the number of reads was greatest at the DS despite the fact the DNA replication does not consistently initiate from DS in Raji genomes [21,22]. The DS peak extended into the adjacent Rep* region, suggesting that these auxillary EBNA1 binding sites contribute to the overall signal observed at the DS region [23]. Importantly, these results provide validation that EBNA1 ChIP Seq analysis was consistent with previous biochemical and genetic studies.

**Table 1:** Solexa Sequencing and Genome Mapping Summary

**Sample**	**Solexa Illumina Pass Filtered sequence**	**Mapped to Human Genome**	**Mapped to EBV Genome**	**Unmapped**
				
EBNA1	14268722	10783205(75.57%)	123764(0.87%)	3361753(23.56%)
IgG	11961444	8317994(69.54%)	35991(0.30%)	3607459(30.16%)

**Figure 2:**
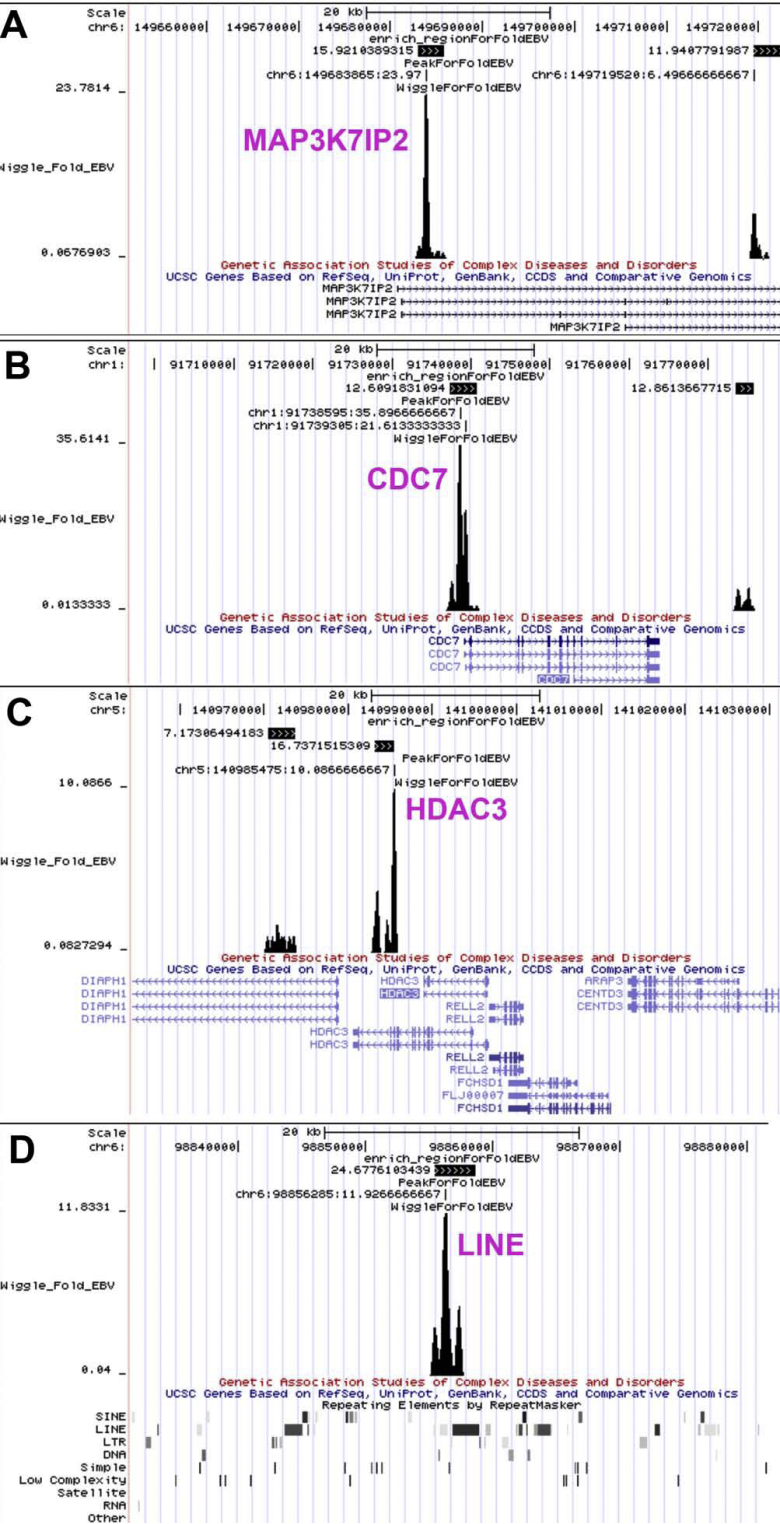
**Example of ChIP Seq data for EBNA1 binding near transcriptional start sites of cellular genes and to a LINE 1 element**. The UCSC browser was used to map EBNA1 peaks, enrichment beds, and Wiggle files to cellular genes for (A) MAP3K7IP2, (B) CDC7, (C) HDAC3, and (D) a LINE1 repeat. RefSeq annotated transcripts are indicated below each wiggle file.

**Figure 3:**
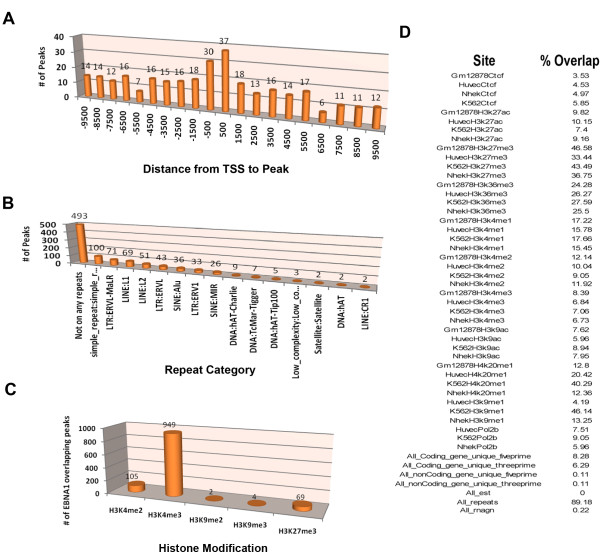
**Summary of EBNA1 binding site overlap with annotated genome landmarks**. The 903 EBNA1 peaks that were filtered for high-occupancy (> 10 fold enrichment and peak scores >8) were analyzed for overlap with annotated genomic features. A) EBNA1 binding sites (# of high occupancy peaks) were analyzed for overlap of RefSeq annotated transcription start sites using windows of 500 bp, as indicated in the X-axis. B) EBNA1 peaks were analyzed for overlap with RefSeq annotations for repetitive DNA elements. Of the 903 total EBNA1 peaks, 410 mapped to repetitive DNA (~45%). Overlaps with various repeats, including LTR, LINE, and SINE elements, are indicated. C) Overlap of EBNA1 with published ChIP-Seq data for histone modifications H3K4me2, H3K4me3, H3K9me2, H3K9me3, and H3K27me3. D) Overlap of EBNA1 binding sites with UCSC annotated binding sites for CTCF and other histone modifications using ChIP-ChIP data sets.

Initial inspection of EBNA1 binding sites across the human genome revealed a large number of candidate sites (4785 total sites with 903 showing >10 fold enrichment over IgG and peak score >8) with various positions relative to transcription start sites. Among the most remarkable was a cluster of highly enriched EBNA1 binding sites extending over ~40 kb region in chromosome 11, within the intergenic region upstream of the divergent promoters for the FAM55 D and FAM55B genes (Figure 1B and 1C). Numerous smaller peaks of EBNA1 binding were found in close proximity to the start sites of many cellular genes (e.g. MAP3K7IP2 and CDC7), as well at alternative promoter start sites (e.g. HDAC3), and repetitive elements (e.g. LINES) as shown in Figure 2. The density of EBNA1 peaks relative to transcription start sites was calculated (Figure 3A). We found that EBNA1 binding sites with 10 fold enrichment relative to IgG were elevated ~3 fold at the positions -500 to +500 relative to transcription start sites. This is consistent with the reported role of EBNA1 in the regulation of cellular gene expression. EBNA1 binding sites were also analyzed for overlap with repetitive DNA elements (Figure 3B). Over 50% of EBNA1 binding sites overlap with a repetitive element. LINE elements were the most prevalent sites of overlap (Figure 2D and 3B). We also found that EBNA1 was enriched ~2-3 fold at telomere repeat DNA (data not shown). This was intriguing since other studies have found evidence for biochemical interactions between EBNA1 and telomere repeat binding factors, as well as the incorporation of telomere repeat DNA into the DS region of OriP [24]. We also examined EBNA1 binding sites for overlap with reported histone modification patterns in lymphoblastoid and fibroblast cell lines from published ChIP-Seq (Figure 3C) and ChIP-ChIP (Figure 3D) data sets. We found that EBNA1 binding sites are predicted to overlap with major peaks of H3K4me3 (Figure 3C), but also with broader regions enriched in histone H3 K27me3, H4K20me1, and H3K9me1 (Figure 3D).

### Identification of cellular EBNA1 binding sites in chromosome 11 and MAP3K7IP2 promoter region

**Figure 4:**
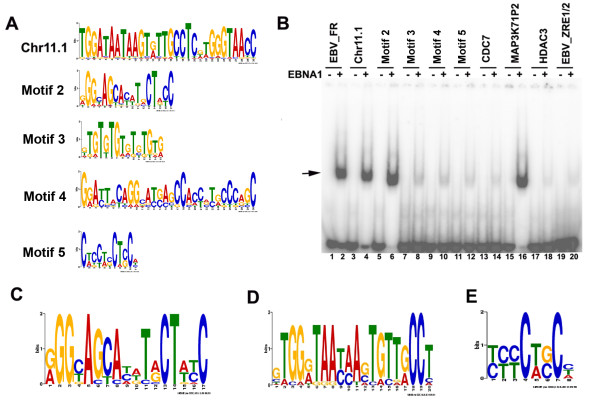
**Consensus binding site of EBNA1 at the Chromosome 11 cluster**. A) MEME and Web Logo analysis of motifs identified in the EBNA1 ChIP-Seq peaks. Chr11.1 represents the motif found in the chromosome 11 cluster, while other Motifs (2-5) were scattered throughout the genome. B) EMSA analysis of ^32 ^P labeled probes containing the EBNA1 peak sites in EBV FR (lanes 1-2), Chr 11.1 (lanes 3-4), Motif 2 (lanes 5-6), Motif 3 (lanes 7-8), Motif 4 (lanes 9-10), Motif 5 (lanes 11-12), CDC7 (lanes 13-14), MAP3K7IP2 (lanes 15-16), HDAC3 (lanes 17-18), or control EBV ZRE1/2 (lanes 19-20) with (+) or without (-) EBNA1 DBD proteins. Arrow indicates bound form of EBNA1. C) Most frequently observed consensus motif derived from 903 cellular binding sites using a 20 bp window. D) Most frequent consensus observed in chromosome 11 repeat using a 20 bp window. E) Most frequent motif overlapping EBNA1 binding sites using a 10 bp window.

To determine if some of the EBNA1 ChIP-Seq sites were bound directly by EBNA1, we assayed the ability of purified EBNA1 protein DNA binding domain (DBD) to bind candidate sequences *in vitro *using EMSA (Figure 4). The high occupancy EBNA1 binding sites throughout the genome (> 10 fold enrichment and peak score >8) were analyzed using the MEME web application http://meme.nbcr.net/meme4_3_0/cgi-bin/meme.cgi. Several candidate motifs are shown in Web LOGO format (Figure 4A), and the most common sequences were synthesized as 40 bp probes for use in EMSA. As a positive control, we assayed EBNA1 DBD for binding to the EBV FR DNA (Figure 4B, lanes 1-2). The most significant pattern found was a motif (Chr11.1) that was repeated 41 times in the chromosome 11 cluster. Other significant motifs (Motifs 2-5) were found scattered throughout the genome. We found that EBNA1 DBD bound with relatively high affinity to the Chr11.1 and Motif 2 (Figure 4B, lanes 2-6), but not to motifs Motifs 3, 4, or 5. We also analyzed the peak sequences enriched in ChIP Seq analysis at the CDC7, MAP3K7IP2, and HDAC3 binding sites (Figure 4B, lanes 13-18). Surprisingly, we found that only the MAP3K7IP2 binding site bound EBNA1 DBD directly. Other sites bound with affinities similar to that of a non-specific control for the EBV ZRE1/2 binding element (Figure 4B, lanes 19-20). The finding suggest that many of the EBNA1 peaks in ChIP Seq are either bound indirectly by EBNA1, or are not centered on the specific DNA recognition site bound by DNA.

To determine if EBNA1 bound to several distinct motifs, we rederived the consensus sites for Motif 2 (Figure 4C) and Chr 11 (Figure 4D) using a higher stringency for peak scores >10 and narrower window. We find that these consensus motifs are significantly different from each other and from previously established binding site consensus from EBV genome sites. The chr11 motif is found 771 times in the complete human genome, but is occupied by EBNA1 at only 23 of these sites (> 8 fold enrichment and peak score > 10). Motif 2 is found 429331 times in the human genome, but is occupied by EBNA1 at only 74 sites. These finding indicate that EBNA1 can bind directly to multiple cellular sites in the cellular genome, but actual binding may be restricted by chromatin context. These findings also indicate that EBNA1 can recognize a more degenerate DNA consensus site than previously appreciated. A similar conclusion was reached by Dresang et al. [17]. We also found that many EBNA1 ChIP-Seq binding sites were enriched for motifs that could not bind EBNA1. Among the most significant consensus motifs that did not bind directly to EBNA1 is shown in Figure 4E. Using search algorithms JASPER and TomTom to identify potential overlapping transcription factor recognition motifs, we found that Motif 4 contains a consensus Sp1 (p value .0011) and a Staf/Znf123 (p value .0023) recognition site. The identification of such consensus sites may help to identify cellular factors that mediate EBNA1 interaction with chromosomes through indirect mechanisms.

### Validation of EBNA1 ChIP binding sites

**Figure 5:**
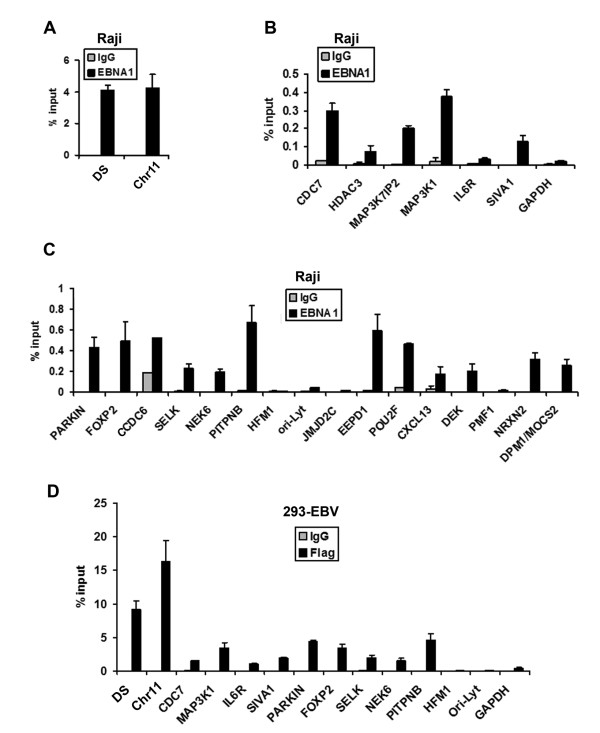
**Real-time PCR validation of ChIP-Seq data for EBNA1 binding sites near transcription starts**. EBNA1 (black bars) or control IgG (grey bars) were assayed by ChIP in Raji cells for DNA at the EBV DS or cellular chromosome 11 cluster (A), the peaks found at the transcription start sites for CDC7, HDAC3, MAP3K7IP2, MAP3K1, IL6R, SIVA1, or negative control GAPDH (B), or EBNA1 peaks within genes for PARKIN, FOXP2, CDC6, SELK, NEK6, PITPNB, HFM1, EBV-OriLyt, JMJD2C, EEPD1, POU2F, CXCL13, DEK, PMF1, NRXN2, or DPM1/MOCS2. D) 293-EBV cells were transfected with FLAG-EBNA1 and assayed by ChIP with antibodies to FLAG (black bars) or IgG (grey bars) at the EBV DS, or cellular chromosome 11, CDC7, MAP3K1, IL6R, SIVA1, PARKIN, FOXP2, SELK, NEK6, PITPNB, HFM1, or negative controls for EBV-OriLyt, or cellular GAPDH.

To determine what percent of the EBNA1 binding sites determined by ChIP-Seq could be validated by independent ChIP analysis using real-time PCR methods, we assayed 26 independent loci that had varying enrichment signals in ChIP-Seq analysis. As expected, EBNA1 was highly enriched at DS (~4% of input DNA was recovered). Interestingly, a similar enrichment was found for the chromosome 11 cluster (Figure 5A). Almost all of the sites enriched by ChIP-seq were similarly enriched by real-time PCR relative to IgG. Several regions were not enriched, including those for EBV OriLyt, and cellular sites for GAPDH, HFM1, PMF1, and IL6R, which had low enrichment (< 10 fold) in ChIP Seq analysis (Figure 5B and 5C). To determine if EBNA1 enrichment was independent of the monoclonal antibody and the cell type, we assayed the binding of FLAG-EBNA1 after ectopic expression in EBV-293 cells (Figure 5D). We found that FLAG-EBNA1 bound with similar pattern and percent enrichment in Flag-EBNA1 transfected cells as did endogenous EBNA1 in Raji cells (Figure 5C). Similar results were also obtained in EBV positive lymphoblastoid cell lines (LCLs) (data not shown). This indicates that our results were not an artifact of the antibody to EBNA1 and not unique to Raji cells.

### Regulation of cellular gene expression by EBNA1

**Figure 6:**
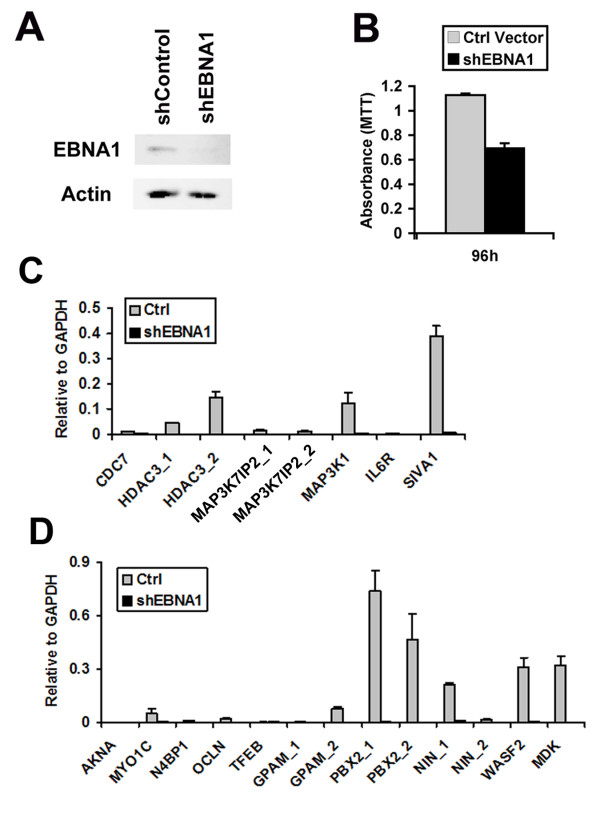
**shRNA depletion of EBNA1 causes a loss of transcription of several genes with EBNA1 binding sites**. A) Western blot showing EBNA1 (top panel) and loading control Actin (lower panel) in Raji cells transfected with plasmid expressing shControl or shEBNA1. B) Raji cells transfected with shControl or shEBNA1 plasmids were selected by GFP positive FACS, and then assayed at 96 hrs post-infection for absorbance in the presence of metabolic activity indicator MTT. C) shControl (grey) or shEBNA1 (black) infected Raji cells were assayed by RT-PCR for genes CDC7, HDAC3, MAP3K7IP1, MAP3K, IL6R, or SIVA1. D) Same as in panel C, except at different cellular genes, as indicated in the legend. For genes with more than one promoter start site, additional primer pairs were used to measure each alternative transcript, as indicated by _1 or _2. All RT-PCR was normalized with GAPDH.

**Figure 7:**
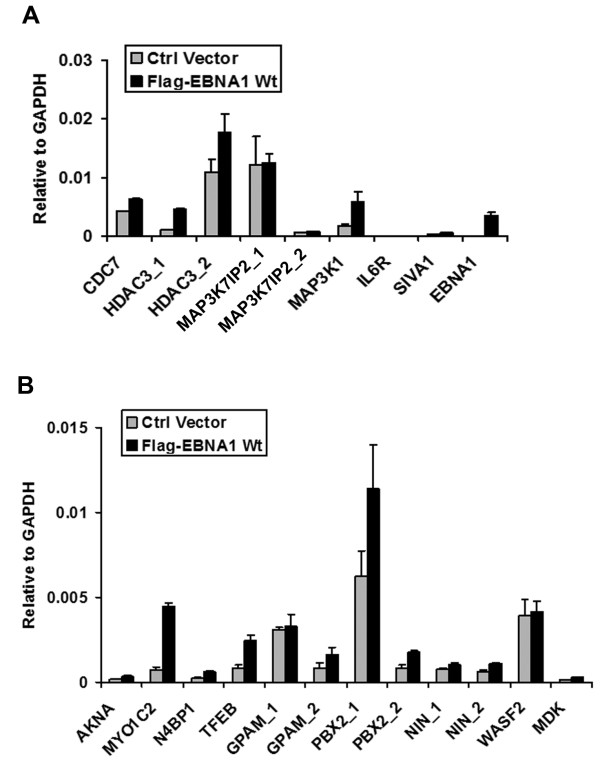
**Ectopic expression of EBNA1 activates a subset of genes with EBNA1 binding sites**. EBV negative Burkitt lymphoma cell line DG75 was transfected with Control vector (grey bars) or with FLAG-EBNA1 (black bars) expression vector and than assayed 48 hrs post-transfection by RT-PCR for A) CDC7, HDAC3, MAP3K7IP2, MAP3K1, IL6R, SIVA1, or control EBNA1, and for B) AKNA, MYO1C, N4BP1, TFEB, GPAM, PBX2, NIN, WASF2, and MDK, as indicated.

To determine if cellular genes containing EBNA1 binding sites near the transcriptional start site were regulated by EBNA1, we assayed the effect of EBNA1 shRNA depletion. Raji cells were transfected with a plasmid expressing shEBNA1 or control shRNA (shControl), along with a GFP marker gene, and then selected by FACS for transfected cells (Figure 6). Western blot analysis indicated that EBNA1 levels were reduced to ~40% of control levels (Figure 6A) at 96 hrs post-infection. Since EBNA1 is required for Raji cell viability, we also observed a ~2 fold reduction in cell metabolic activity as measured by MTT assay (Figure 6B). To determine if EBNA1 depletion altered gene expression of any of the EBNA1 bound genes, we compared the RNA levels of several candidate genes by RT-PCR (Figure 6C and 6D). For genes with documented alternative promoter start sites, we generated primer pairs that would detect initiation at both transcription start sites. We found that EBNA1 depletion caused a significant reduction of several mRNAs, including HDAC3, MAP3K1, SIVA1, MYO1C, PBX2, NIN (uc001wyk), WASF2, and MDK. We did not find any genes that were upregulated by EBNA1 depletion, suggesting that EBNA1 does not function as a general transcriptional repressor of these tested genes in Raji cells. To further test the role of EBNA1 in cellular gene regulation, we assayed the ability of transiently transfected FLAG-EBNA1 to alter cellular gene transcription in an EBV negative Burkitt lymphoma cell line DG75 (Figure 7). Using this approach, we found that FLAG-EBNA1 transfection stimulated expression of CDC7, HDAC3, MAP3K1, MYO1C, TFEB, and PBX2. RT-PCR of EBNA1 mRNA was used as a positive control for EBNA1 expression. These results suggest that EBNA1 can activate a subset of genes when ectopically expressed in EBV negative Burkitt lymphoma cell lines.

### Histone modifications at EBNA1 binding sites

**Figure 8:**
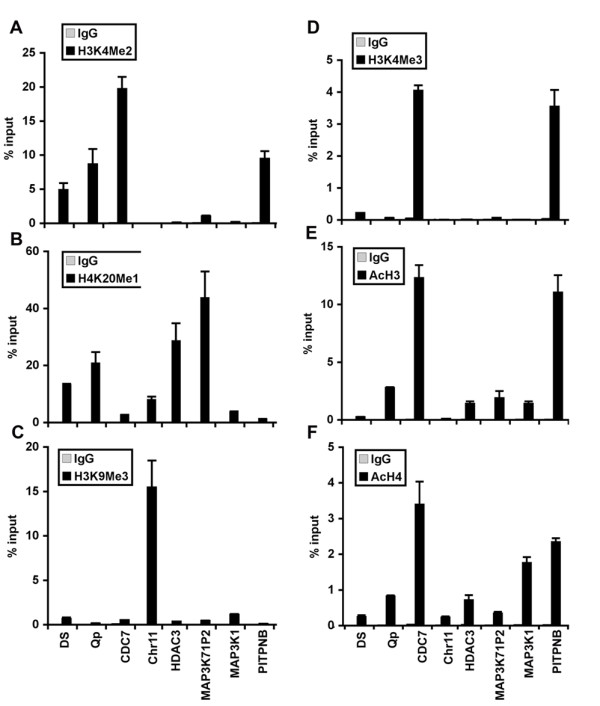
**Histone modifications associated with EBNA1 binding sites**. Raji cells were assayed by ChIP using control IgG (greay bars) or modification-specific antibodies (black bars) for (A) H3K4me2, (B) H4K20me1, (C) H3K9me3, (D) H3K4me3, (E) AcH3, and (F) AcH4 at EBNA1 binding sites in EBV DS and Qp, or cellular CDC7, Chr11, HDAC3, MAP3K7IP2, MAP3K1 or PITPNB. ChIP DNA was quantified by real-time PCR as percent of input DNA.

To explore the possibility that EBNA1 may associate with chromatin enriched in a particular histone tail modification, we first assayed the overall correlations of EBNA1 binding sites with reported histone tail modifications in human lymphoid cells (Figure 3C and 3D). Based on this first analysis, we selected a set of histone tail modification-specific antibodies for ChIP assays at several EBNA1 binding sites identified in Raji cells (Figure8). We first assayed histone H3K4me2, which has been previously reported to be elevated at EBNA1 binding sites in the EBV genome [25]. As expected, we found that H3K4me2 was highly elevated at DS and Qp in the EBV genome (Figure 8A). H3K4me2 was also elevated at the cellular EBNA1 binding sites associated with CDC7 and PTPNB. Histone H4K20me1 was found to have a relatively high genome-wide correlation with EBNA1 binding (Figure 3D), and was indeed elevated at DS and Qp, as well as at the cellular EBNA1 binding sites associated with CDC7, Chr11, HDAC3, MAP3K7IP2, and MAP3K1 (Figure 8B). Histone H3K9me3, a mark associated with heterochromatin and repetitive DNA, was found to be highly elevated at the Chr11 repeat cluster (Figure 8C). Histone H3K4me3 and acetylated histone H3 (AcH3) and H4 (AcH4), which are associated with euchromatic and transcriptionally active regions, were elevated at cellular genes for CDC7 and PTPNB, while MAP3K1, MAP3K7IP2, and HDAC3 were found enriched just in AcH3 and AcH4 (Figure 8D-F). These findings suggest that EBNA1 binding may correlate with some histone modifications, but in a manner that is complex and context-dependent.

### EBNA1 binding site close to the cMyc-IgG translocation break point in Raji Burkitt Lymphoma

Raji has a rearranged copy of the c-myc gene adjacent to the gamma 1 constant region gene of the human immunoglobulin heavy-chain locus, t(8;14) (q24;q32) [26]. Examination of EBNA1 binding sites in these translocated regions revealed peaks of >3 fold enrichment at the cMyc 3' end of chromosome 8 and >10 fold enrichment within the IgH locus of chromosome 14. In Raji Burkitt lymphoma, these two sites are fused together by a breakpoint in the cMyc and IgH 5' region, thus bringing the two EBNA1 binding sites in close proximity in the translocated allele. Although the mechanism of translocation is unknown, EBV has been considered a potential driving force for the Burkitt's translocations, and it is possible that these EBNA1 binding sites may link these sites to facilitate translocation.

## Discussion

### EBNA1 can interact with a large number of cellular binding sites

In this study, we used ChIP-Seq to identify ~903 high occupancy (> 10 fold enrichment and peak score > 8), and ~4300 moderate occupancy (> 3 fold enrichment and peak score >5) binding sites for EBNA1 in the cellular chromosome of a human Burkitt lymphoma cell line. Several (~25) of the high and low occupancy binding sites identified by ChIP-Seq were validated for binding by conventional ChIP and real-time PCR (Figure 5). There was a good correlation between ChIP-Seq and ChIP-PCR for these binding sites, providing a high level of confidence in the ChIP-Seq data. Furthermore, only bonafide EBNA1 binding sites in the EBV genome (FR, DS, and Qp) scored positive in ChIP-Seq analysis (Figure 1A), suggesting that few false positives were generated by this method. Among the high-occupancy binding sites, we noted that ~7% are located within 500 bp of an annotated or predicted transcription start site (Figure 3A). We also noted that ~45% of EBNA1 binding sites overlapped with a repetitive DNA element (Figure 3B). Several DNA motifs could be identified in the high-occupancy EBNA1 binding site data set, but only two of these were found to bind directly to recombinant EBNA1 protein in vitro (Figure 4). Since EBNA1 is known to bind DNA directly through its carboxy-terminal DNA binding domain, and indirectly through its amino terminal tethering domain, it seems likely that many of the binding sites identified by ChIP-Seq represent a composite of these direct and indirect DNA-binding modes of EBNA1.

### Identification of high affinity cellular binding sites

A remarkable finding from this study was the identification of a cluster of high-affinity EBNA1 binding sites in chromosome 11. The cluster represents ~10 kb of repetitive sequence situated between the divergent promoters for the Fam55B and Fam55 D genes. The function of the Fam55B and D proteins is not known, and shRNA depletion of EBNA1 had no detectable effect on Fam55B or D gene transcription. This region was elevated in histone H3 K9me3 (Figure 8) suggesting that it is largely heterochromatic and unlikely to be involved in transcription activation. We considered whether this site may represent a cellular origin of DNA replication, but we were unable to identify ORC2 or MCM protein binding at this site (data not shown). Purified EBNA1 DBD protein bound with high affinity to the major repeat elements in the chromosome 11 cluster, indicating that the binding is direct and mediated by the EBNA1 DNA binding domain. At present, it is not clear whether EBNA1 binding to this region of chromosome 11 has any functional significance.

### Novel EBNA1 binding sites

Position weighted matrix (PWM) analysis and Web LOGO presentation revealed that many cellular EBNA1 binding sites are distinct from the consensus sites observed at EBV genome binding sites found at the FR, DS, or Qp regions. The chromosome 11 binding site consensus TGG[g/a]TAA[T/C][A/C]A[g/c]TGTT[G/A]CCT and the Motif 2 GG[C/T]AGCAtaT[A/G]CT[A/T][T/C]C do not resemble the consensus derived for previously known EBNA1 binding sites in the viral genome. However, our Motif 2 is similar the new consensus G[A/G][T/C]AGcATaTGCTaCC derived by Dresang et al using 70 viral and cellular binding sites [17]. In a separate study, Canaan et al. identified GaA[G/A]TAT[T/C] as a consensus site for EBNA1 binding at cellular genes subject to EBNA1 dependent regulation and association with EBNA1 protein by ChIP [18]. However, it was not clear from the Canaan et al. study whether these binding sites are bound directly or indirectly by the EBNA1 DNA binding domain. We also identified several motifs enriched in the EBNA1 ChIP-Seq peaks that did not bind directly to EBNA1 DNA binding domain *in vitro*. These sites may reflect indirect DNA binding by EBNA1, potentially through interactions with other sequence specific factors or chromatin-associated proteins. Cellular factors that have been implicated in mediating EBNA1 tethering to metaphase chromosomes, including EBP2 [27-29], histone H1 [16], and HMGA1 [14,30], may be good candidates for interactions with some of these indirect binding motifs. In the future, it will be important to determine if there are functional differences between these different classes of EBNA1 binding sites, and what cellular factors mediate the indirect binding of EBNA1 to cellular chromosomes.

### EBNA1 regulated cellular genes

Ectopic expression of EBNA1 has been shown to regulate several cellular genes, including NOX2 in Ramos [19], and the chemokines CCL4, CCL3, and CCL18 in BJAB cells [18]. Dresang et al. identified and confirmed direct binding of EBNA1 to several cellular promoters, but could not confirm that these were indeed EBNA1-responsive promoters in plasmid based reporter assays [17]. These previous studies identified ~366 cellular genes as potential targets of EBNA1. In our study, we considered only 36 candidate cellular genes that have highly enriched (peak score > 8 and enrichment >10) EBNA1 binding sites within 500 bp of the transcriptional start site (Table 2). Of these 36, only four genes were common to the previous studies, namely MOCS3, CDC7, TTC7A, and FGB. However, lower threshold EBNA1 (peak score > 3 and enrichment >3) binding sites were observed at many of the sites identified by Dresang et al., including binding sites upstream of SELK, IL6R, MKI67, and MYO5B. We did not find significant enrichment of EBNA1 at NOX2 or CCL4. Some of these discrepancies may be a result of differences in cell types used for each experiment. Different cell types, as well as differentiation states, may restrict EBNA1 binding through changes in chromatin structure or epigenetic modifications. We found some evidence that EBNA1 binding sites correlated with histone modifications, namely the euchromatin-associated H3K4me3 and H3K4me2 (Figure 3C). However, this analysis is limited since the histone modifications were analyzed in EBNA1 negative T-cells [31], and not in the same Raji cells as EBNA1 ChIP Seq experiments were performed. A small-scale analysis of confirmed EBNA1 binding sites in Raji cells revealed a complex correlation with histone modifications (Figure 8), suggesting that EBNA1 can bind to various chromatin structures. However, additional studies of EBNA1 and histone modification co-occupancy are required to determine whether EBNA1 binding sites have a common chromatin environment.

**Table 2:** EBNA1 binding sites close to RefSeq genes

Chr	Start	End	Motif	RefSeq gene hit		Dist. to hit
chr1	52074635	52074636	Motif 5	NR_031580	MIR761	500
chr1	91738595	91738596	Motif 3	NM_001134419	CDC7	500
chr1	91739305	91739306	Motif 5	NM_001134420	CDC7	500
chr1	154170655	154170656		NM_014949	KIAA0907	500
chr10	61338790	61338791	Motif 2	NM_005436	CCDC6	3000
chr11	6421695	6421696		NM_000613	HPX	4000
chr12	67007430	67007431		NM_017440	MDM1	5000
chr12	108693660	108693661	Motif 2	NR_026661	MGC14436	4000
chr13	35769560	35769561	Motif 2	NM_001144985	C13orf38	500
chr13	35769000	35769001		NM_001144985	C13orf38	2000
chr14	50358425	50358426	Motif 5	NM_016350	NIN	3000
chr14	23969975	23969976	Motif 4.Motif 5	NM_015299	KHNYN	2000
chr14	104287630	104287631	Motif 5	NM_021709	SIVA1	4000
chr15	26999285	26999286		NM_001130414	APBA2	3000
chr16	3222305	3222306		NM_001145447	ZNF200	4000
chr16	3221595	3221596		NM_001145447	ZNF200	4000
chr17	71770850	71770851	Motif 2	NM_182565	FAM100B	4000
chr17	1335740	1335741	Motif 4	NM_001080950	MYO1C	500
chr17	58917775	58917776	Motif 2.Motif 5	NM_152830	ACE	3000
chr17	64015725	64015726		NM_212471	PRKAR1A	4000
chr2	190751405	190751406	Motif 5	NM_001042519	C2orf88	4000
chr20	49008975	49008976	Motif 2	NM_014484	MOCS3	500
chr20	36872330	36872331	Motif 2.Motif 5	NM_015568	PPP1R16B	5000
chr22	26645510	26645511		NR_026962	LOC284900	500
chr3	75761660	75761661		NR_031714	MIR1324	2000
chr6	18376590	18376591		NM_001134709	DEK	4000
chr6	149683865	149683866	Motif 5	NM_015093	MAP3K7IP2	4000
chr6	39190140	39190141		NM_018322	C6orf64	2000
chrX	41428495	41428496	Motif 2	NM_001097579	GPR34	5000

Each hit peak in the ChIP-seq enrichment list was labeled with our consensus motifs that lay within 50 nts upstream or downstream of the peak site. This annotated dataset was then used to search for overlaps with a list of RefSeq genes downloaded from the UCSC table browser. This overlap search was done with a Python script from Priyankara Wickramasinghe (Wistar Institute). Genes whose TSS lie within a distance of 500, 1000, 2000, 3000, 4000, and 5000 nt from EBNA1 ChIP-seq peaks were subsequently annotated on to the annotated hit peak list. The Galaxy suite http://main.g2.bx.psu.edu/ was used to combine and organize the various data lists in each step.

### EBNA1 may regulate to chromosome structure

**Figure 9:**
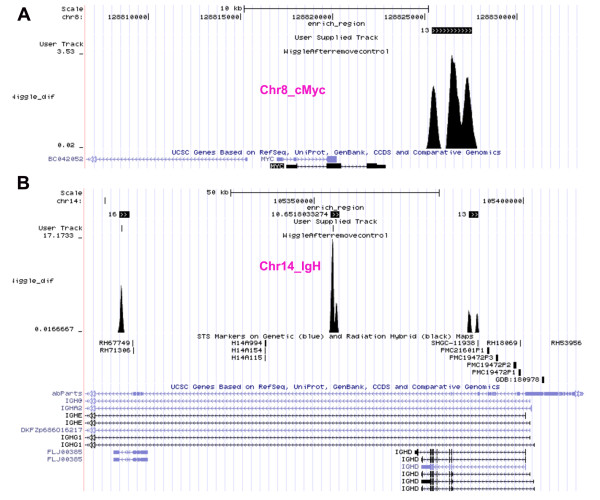
**EBNA1 binding site near the point of cMyc-IgG translocation in Raji Burkitt lymphoma cells**. EBNA1 wiggle tracks and peak bed enrichment from UCSC browser of the translocation regions at the chromosome 8 cMyc locus (A) and the chromosome 14 IgH gene locus (B).

The large number of direct and indirect EBNA1 binding sites distal to transcription start sites suggests that EBNA1 may be involved in modulating chromosome structure. EBNA1 is known to mediate long-distance interactions important for transcription of Cp during EBV infection of primary B-cells [8]. EBNA1 is also known to form highly stable homotypic interactions through its linking domains [32], which are thought to play important roles in tethering EBV episomes to metaphase chromosomes [33]. EBNA1 has also been a suspect in EBV-associated lymphomagenesis, especially Burkitt lymphoma where EBNA1 is expressed early and consistently throughout cancer cell evolution. Our ChIP-Seq data revealed that EBNA1 can bind to regions close to the chromosomal translocation break-points in both cMyc and IgG heavy chain enhancer regions (Figure 9), which represents the defining translocation associated with Burkitt's lymphoma. This provides a potential mechanism for EBNA1 in facilitating chromosome translocation by potentially mediating an interaction between these two loci. Further studies will be required to determine whether EBNA1 binding can mediate interactions between these two chromosomal sites in Burkitt's and non-Burkitt's lymphoma cells, and whether EBNA1 mediates long-distance interactions between other cellular and viral chromosomal sites identified in this study.

## Methods

### Cells and Plasmids

Raji cells (human EBV positive Burkitt lymphoma line) and DG75 (human EBV negative Burkitt lymphoma line) were maintained in RPMI containing 10% FBS and supplemented with glutamax (Invtitrogen) and antibiotics (penicillin and streptomycin). EBV-293 contains a hygromycin resistant EBV bacmid in human embryonic kidney (HEK) 293 cells (a kind gift of H. Delecluse) were maintained in RPMI containing 10% FBS, hygromycin (100 μg/ml), glutamax, and antibiotics. pCMV-Flag-EBNA1 was previously described [15]. shRNA directed against EBNA1 was generated by cloning the targeting hairpin sequence (gatatgtctcccctccctcctaggccactcaagcttcaatggcctaggagagaagggagacacatc) into the pENTR/D-Topo vector (Invitrogen).

### Chromatin immunoprecipitation (ChIP) Assays

ChIP assays were performed as described previously [34]. Quantification of precipitated DNA was determined using real-time PCR and the standard curve method for absolute quantitation (ABI 7000 Real-Time PCR System). IPs were performed in triplicate for each antibody and the PCR reactions were repeated at least three times and standard deviations were indicated by error bars. Primers for ChIP assays are listed in Additional File 1 in the online Data Supplement. The following rabbit polyclonal antibodies were used for ChIP assays: anti-EBNA1 (305/10 wk), anti-IgG (Santa Cruz Biotechnology), anti-Flag (Sigma), anti-Acetylated histone H3 and H4 (Millipore), anti-dimethylated histone H3K4 (Abcam), anti monomethylated H4k20 (Abcam), and anti-trimethyl histone H3K9 and H3K4 (Millipore). Mouse monoclonal anti-actin (Sigma) and anti-EBNA1 (Advance Biotechnology) were used for Western Blotting.

### Quantitative RT-PCR

Briefly, RNA was isolated from 2 × 10^5 ^cells using RNeasy Kit (Qiagen) and then further treated with DNase I. Reverse transcriptase PCR (RT-PCR) was done as previously described. Real-time PCR was performed with SYBR green probe in an ABI Prism 7000 according to the manufacturer's specified parameters. Primer sequences for RT-PCR are listed in Additional File 2.

### MTT assay

1 × 10^4 ^Raji cells were plated in 96-well plates at 96 hrs post-transfection of shEBNA1 or Control shRNA. Cell viability was then measured by incorporation of 3-(4,5-dimethylthiazol-2-yl)-2,5-diphenyl tetrazolium bromide (MTT) (Millipore, Cell Growth Assay Kit), according to the manufacturer's protocol.

### EMSA

Purified EBNA1 DNA binding domain (DBD) (aa 459-607) was expressed and purified from E. coli as a hexa-histidine fusion protein in *Escherichia coli*.

Protein-DNA binding reactions contained 10% Glycerol, 200 mM NaCl, 20 mM Tris-Cl pH 7.4, 1 mM DTT, 10 μg/mL BSA, 10 nM ^32^P-labeled oligonucleotide DNA and 246 nM purified EBNA1 DBD. Samples were incubated for 20 min at 25°C then loaded onto a 6% polyacrylamide gel and electrophoresed for 90 minutes at 170 V in 1× TBE. Gels were dried and visualized by PhosphorImager. Oligonucleotides used for EMSA are listed in Additional File 3.

### Chromatin-Immunoprecipitation for High Throughput Sequencing (ChIP-Seq)

Solexa ChIP-Seq experiments were performed with 2 × 10^6 ^Raji cells per IP with either EBNA1 monoclonal antibody or control mouse IgG. ChIP methods were identical to conventional ChIP assays [34] with the exception that competitor salmon sperm DNA was excluded from all IP and wash buffers, and purified ChIP DNA was resuspended in 25 dH_2_O. DNA fragments of ~150-300 bp range were isolated by agarose gel purification, ligated to primers, and then subject to Solexa sequencing using manufacturers recommendations (Illumina, Inc.).

### Bioinformatics Analysis of ChIP-Seq

#### Sequencing

Image analysis and base calling of ChIP-seq data was performed using Illumina pipeline software version 1.4. Sequence alignment to the human genome hg18 was done using Illumina casava_1.4 module. Uniquely aligned sequence tags, with up to two mismatches, were taken for the downstream analysis.

#### Peak Calling

A combination of fold ratio and Poisson model for the tag distribution [35] was used to define peaks as follows: (i) Identification of genomic regions (of length 1000 bp) enriched with ChIP-seq sequence tags using fold ratio - A genomic region is considered as sequence enriched if the fold ratio, calculated using number of reads normalized to the total reads within that region in ChIP (antibody treatment) sample divided by the number of reads normalized to the total reads in control sample (IgG control) in the same region, is higher than the given cutoff. Nearby enriched regions were merged to make broader enriched genomic regions. A cutoff of 3 was applied to find the initial genomic regions of enrichment at this stage. (ii) Creating the read overlapping profile for each identified region from step 1, by extending the sequence reads from the 5' end to the 3' end of the reads up to 300 bps (the average length of the ChIP-DNA fragment sequenced from the Solexa GA with Illumina standard ChIP-seq protocol) for the experiment sample. (iii) Peak identification, by using Poisson model - by counting the number of overlapped reads at each nucleotide position and defining the genomic position with the highest number as the peak position within the significant region. Finally, only those genomic regions that have fold ration > 10 and peak score > 8 and p value < 0.001 (as determined by Poisson background model) are considered as statistically significant. Peak score is calculated as the average value of raw counts within a given region of significant fold enrichment relative to control IgG levels. The average is measured for overlapping tags at every base after extending the tags to their average tag length within the significant region.

#### Overlapping of TSS with Peak

For annotating the ChIP-seq peaks, we referred to gene information tracks from various sources available at UCSC genome browser. The tracks include Refseq gene, UCSC gene, Ensembl gene, and Vega gene. Every peak was annotated to the closest TSS regardless whether the peak is residing upstream or downstream to the TSS. Figure 3A shows the distribution of ChIP-seq peaks relative to the TSSs. We then selected a subset of ChIP-seq peaks, such that the peaks are within ± 500 bp around TSS. We call these peaks as TSS associated peaks. Overlap with specific genes is provided in Table 2.

#### Overlapping of repeats with Peak

Repeat region files were downloaded from UCSC genome browser. All those peaks that fall in repetitive regions are annotated according to the type of repetitive region. Same method was used in finding the overlap of peaks with the repeat sub categories.

#### EBNA1 binding Motif Identification

We selected only the highly enriched genomic regions (enriched region fold ratio > 10 and peak score > 8) for motif identification. A sequence window of 60 bp around each peak was used for motif searching. We applied MEME online version to find the statistically significant sequence motifs [36,37]http://meme.nbcr.net/meme4_4_0/intro.html. Possible EBNA1 binding motifs were predicted based on highest number of occurrence with the lowest p-value under "zero or one per sequence" option. Position weighted natrix (PWM) generated by MEME were then represented in the logo format by using Web Logo http://weblogo.berkeley.edu/logo.cgi to generate consensus sequences for multiple cellular EBNA1 binding sites. PWMs of the motifs identified by MEME were matched with the JASPER core database http://jaspar.genereg.net/ using the TOMTOM program http://meme.nbcr.net/meme4_3_0/cgi-bin/tomtom.cgi with a q-value significance (false discovery rate) threshold of under 0.5.

#### Overlap of histone modification and EBNA1 peaks

H3K4me2, me3 and H3K9me2, me3 ChIP-seq datasets were downloaded from NCBI GEO and SRA databases, published in [31,38]. The accession numbers for the datasets are as follow: SRX000147, SRX000148, SRX000153, SRX000154, and GSM325898. Each ChIP-seq dataset was processed at p < 0.001 using Poisson background model. A histone modification mark is considered as overlapping with EBNA1 peak if it falls within ± 1000 bp around the EBNA1 peak.

## Competing interests

The authors declare that they have no competing interests.

## Authors' contributions

FL performed all experiments shown in Figures 5, 6, 7, 8. PW performed bioinformatics analyses of ChIP-Seq. JN performed the ChIP-Seq experiment. KT identified consensus binding sites and bioinformatics analysis of trancription factors. PW performed EMSA analysis. LS provided Solexa sequencing. RD provided bioinformatic support and programming for ChIP Seq analysis. PL designed experiments, interpreted results, and wrote the manuscript.

## Supplementary Material

Additional file 1 **Primers usee for validation of ChIP-Seq**. Click here for file

Additional file 2 **Primers used for RT-PCR**. Click here for file

Additional file 3 **Primers used for EMSA probes**. Click here for file
