# Supplementary material for: Genome-wide analysis of host-chromosome binding sites for Epstein-Barr Virus Nuclear Antigen 1 (EBNA1)
Source: Virol J. 2010 Oct 7;7:262. doi: 10.1186/1743-422X-7-262 (PMC2964674; doi:10.1186/1743-422X-7-262)
Supplement: Additional file 1 — Primers usee for validation of ChIP-Seq. [file 1743-422X-7-262-S1.DOC]

|  | **Primer for ChIP Validation** | **GENE** |
| --- | --- | --- |
|  | ATGTAAATAAAACCGTGACAGCTCAT | DS_5' |
|  | TTACCCAACGGGAAGCATATG | DS_3' |
|  | GAAAAGGCGCGGGATAGC | Qp_5' |
|  | AAAATGCCAAAATGTAAGGATAGCA | Qp_3' |
|  | CGTCTTACTGCCCAGCCTACTC | Ori-Lyt_5' |
|  | AGTGGGAGGGCAGGAAATG | Ori-Lyt_3' |
|  | TGGGCTACACTGAGCACCAG | GAPDH_5' |
|  | GGGTGTCGCTGTTGAAGTCA | GAPDH_3' |
|  | CCTCGTGGGTAAGCACTGTT | Chr11_5' |
|  | CCATGAGGCCACACTTATT | Chr11_3' |
|  | TGGAGTGCCCAATGTGTTGT | CDC7_5' |
|  | TCAGGTGCTGACACATCCCTAT | CDC7_3' |
|  | CCTGGGCTCCACCTTTCA | HDAC3_5' |
|  | GCCAGGTCAGCAGCTCAGA | HDAC3_3' |
|  | GGAATGTGGAGATGTGGGTAGTG | MAP3K7IP2_5' |
|  | TCCTTTGTACAGTTCAGCATGATCA | MAP3K7IP2_3' |
|  | CCAAGCAAGAGAACCATTCGA | MAP3K1_5' |
|  | CCACTTGGGCCTGCTGAT | MAP3K1_3' |
|  | CCCCAATCACGGCTCACT | IL6R_5' |
|  | GAGGTGGGAAAATTGCTTGAGT | IL6R_3' |
|  | ACGGCAGGAGGAGAAAGGA | SIVA1_5' |
|  | CCATGGGTGGCTGAAACCT | SIVA1_3' |
|  | CCACTGCCTGGCTCTTCTCT | PARKIN_5' |
|  | TCTGACCCATTCGAAGATAAGCA | PARKIN_3' |
|  | CAGGGACTCATCTCCATTCCA | FOXP2_5' |
|  | CCTTGAGGCAGCGATTGG | FOXP2_3' |
|  | CACCTCCTTTGGAGAAATAATGAAATT | CCDC6_5' |
|  | TTGTTCATGTGTTTTTGCAGCTT | CCDC6_3' |
|  | CGACGGGCGTTTTTGC | SELK_5' |
|  | CCGCCTCCGCCTAACC | SELK_3' |
|  | GTCCCGAAGGGAAGACGAA | NEK6_5' |
|  | CGAGGGTGCGGATCCA | NEK6_3' |
|  | TCTGGGCAGCCTACGCTTT | PITPNB_5' |
|  | CGCAAAACGGCTTCCAAA | PITPNB_3' |
|  | CCCGCTTTCACGGTCTGTA | HFM1_5' |
|  | GGGCAAAAGCTCGCTTGA | HFM1_3' |
|  | TGGAGGCTGGGCTTGGT | JMJD2C_5' |
|  | GCCTCAGCCTCCCAAAGTG | JMJD2C_3' |
|  | TCCCAGGAGGTGCAAATCTT | EEPD1_5' |
|  | TCAGGGCATAAGTCTGCGTTT | EEPD1_3' |
|  | ACCGAGGACCCTGGTTTCTT | POU2F_5' |
|  | CATGGCAAGGAATGGAGAACA | POU2F_6' |
|  | GCCTGTGCTTCCCAGGAA | CXCL13_5' |
|  | AATTGAGCCATGTTCTCCTCATG | CXCL13_3' |
|  | AAGAGGCATCTGGGTGACACA | DEK_5' |
|  | CTAGATCCCATGTCCCCTCATC | DEK_3' |
|  | TGGGAAGCGGAGGTTTCA | PMF1_5' |
|  | GCCCAGGCTAGAATGCAATG | PMF1_3' |
|  | TGTTGCAGGGTCTGTGATCTG | NRXN2_5' |
|  | TTGCCTCTGGCTAAGGAAGTG | NRXN2_3' |
|  | CCTCAATTCGGCAGTGGAAT | DPM1/MOCS2_5' |
|  | GCAGTGGCTGGCGTAAGG | DPM1/MOCS2_3' |
