# Supplementary material for: Genome-wide analysis of host-chromosome binding sites for Epstein-Barr Virus Nuclear Antigen 1 (EBNA1)
Source: Virol J. 2010 Oct 7;7:262. doi: 10.1186/1743-422X-7-262 (PMC2964674; doi:10.1186/1743-422X-7-262)
Supplement: Additional file 2 — Primers used for RT-PCR. [file 1743-422X-7-262-S2.DOC]

|  | **Primer Sequence for RT-PCR** | **Gene** |  |
| --- | --- | --- | --- |
|  | GAGCACCAGAGGTCTTGACAAA | CDC7_Fwd |  |
|  | TGCAGACCACATGTCAATTGC | CDC7_Rev |  |
|  | CCTTTTCCAGCCGGTTATCA | HDAC3_1_Fwd |  |
|  | ACAATGCACGTGGGTTGGT | HDAC3_1_Rev |  |
|  | GGGCTCTGGCTCCCTAACA | HDAC3_2_Fwd |  |
|  | TCCTCCCCACACTTGAAAACA | HDAC3_2_Rev |  |
|  | GCATTCTGGCTGGGTATCTCA | MAP3K7IP2_1_Fwd |  |
|  | CAGGCTGTGAAGGCTGATAAAC | MAP3K7IP2_1_Rev |  |
|  | AATTCGCTGTGAACAGTGTGAGA | MAP3K7IP2_2_Fwd |  |
|  | GTTTTAGAGAAGATACAGGGCCATTT | MAP3K7IP2_2_Rev |  |
|  | CAAACCGCCGTGTTAACAAA | MAP3K1_Fwd |  |
|  | GGCCCTATCTGCTGCAGTAAGT | MAP3K1__Rev |  |
|  | CAGCAGGGAGGGCTTCTG | IL6R_Fwd |  |
|  | CTGACAACAAACAAAGCTGCAGTA | IL6R_Rev |  |
|  | GACTGCAGTGACATGTACGAGAAA | SIVA1_Fwd |  |
|  | CCAGCCTCAGGTCTCGAACA | SIVA1_Rev |  |
|  | GAGATTGCCTGGTGAGCCTAGA | AKNA_Fwd |  |
|  | TGGCTCGCTGCCTTCCT | AKNA_Rev |  |
|  | GCCCGTCCAGTATTTCAACAA | MYO1C_Fwd |  |
|  | AAATCGAGATGATGCCCTTAAACT | MYO1C_Rev |  |
|  | GCTATGGGAAGGAAGGATTGC | N4BP1_Fwd |  |
|  | GGTTTCGGAAGGAATGATGAATT | N4BP1_Rev |  |
|  | CACACAGGACGTGCCTTCAC | OCLN_Fwd |  |
|  | GAGTATGCCATGGGACTGTCAA | OCLN_Rev |  |
|  | CGGCAGAAGAAAGACAATCACA | TFEB_Fwd |  |
|  | GATGCGGTCATTGATGTTGAA | TFEB_Rev |  |
|  | GAGACGAATTTGCCGCTTC | GPAM_1_Fwd |  |
|  | GAATGAAAGTGAGCAGCAGATAGTCA | GPAM_1_Rev |  |
|  | CGTCCAGTGTGGCGTTGA | GPAM_2_Fwd |  |
|  | GAGCTCCGATCACCTACATTATAATTT | GPAM_2_Rev |  |
|  | CGTGGCCCCCAAAGAGA | PBX2_1_Fwd |  |
|  | TGGATGGCGCTGAACTTTC | PBX2_1_Rev |  |
|  | CTCCGGACGGCTTACTTACCT | PBX2_2_Fwd |  |
|  | CCACCGAGCGATTCTATTGG | PBX2_2_Rev |  |
|  | GATCTGGACAAGGCCGAGAA | NIN_1_Fwd |  |
|  | TCTATGGCCGCATGGTGAT | NIN_1_Rev |  |
|  | GCCTACCCGGTCATCTCTGTT | NIN_2_Fwd |  |
|  | ATGGCAATAAAGGGATGTAAAACTG | NIN_2_Rev |  |
|  | CGTCGCATTGCTGTTGAGTAC | WASF2_Fwd |  |
|  | CCAGTCGTCCTCATCAAATTCA | WASF2_Rev |  |
|  | GGGAGGGACAAGGGATTCTG | MDK_Fwd |  |
|  | GCTCTGGGACTCACATTGCTT | MDK_Rev |  |
|  | GGTCGTGGACGTGGAGAAAA | EBNA1_Fw |  |
|  | GGTGGAGACCCGGATGATG | EBNA1_Rev |  |
