# Supplementary material for: Genome-wide analysis of host-chromosome binding sites for Epstein-Barr Virus Nuclear Antigen 1 (EBNA1)
Source: Virol J. 2010 Oct 7;7:262. doi: 10.1186/1743-422X-7-262 (PMC2964674; doi:10.1186/1743-422X-7-262)
Supplement: Additional file 3 — Primers used for EMSA probes. [file 1743-422X-7-262-S3.DOC]

**Additional File 2**

**Oligonucleotides Used for EMSA Probes**

FR: gatccggatacagattaggatagcatatactaccca.

Chr11.1: tggataataagtgttgcctcttgggtaacc.

Motif 2: atggtagaggcagcacatgctacctaacct.

Motif 3: gtgtgtgtgtgtgtgtgtgtgtgtgtgtgt.

Motif 4: ggattacaggcatgagccaccatgcccagc.

Motif 5: ggatctcctcctccacaccagctgctcagc.

CDC7: tgctaatttctactaacacagccacaaaca.

MAP3K7IP2: ggataggatgtcacatacgaattttgaggg.

HDAC3: cgggtgctgacatctggatgaagtgtgaag.
